# Development of Modified Vaccinia Virus Ankara-Based Vaccines: Advantages and Applications

**DOI:** 10.3390/vaccines10091516

**Published:** 2022-09-13

**Authors:** Olga Vladimirovna Orlova, Dina Viktorovna Glazkova, Elena Vladimirovna Bogoslovskaya, German Alexandrovich Shipulin, Sergey Mikhailovich Yudin

**Affiliations:** Federal State Budgetary Institution “Centre for Strategic Planning and Management of Biomedical Health Risks” of the Federal Medical Biological Agency, 119121 Moscow, Russia

**Keywords:** viral vector vaccines, poxvirus, smallpox vaccine, vaccine development, clinical trial

## Abstract

Modified vaccinia virus Ankara (MVA) is a promising viral vector for vaccine development. MVA is well studied and has been widely used for vaccination against smallpox in Germany. This review describes the history of the origin of the virus and its properties as a vaccine, including a high safety profile. In recent years, MVA has found its place as a vector for the creation of vaccines against various diseases. To date, a large number of vaccine candidates based on the MVA vector have already been developed, many of which have been tested in preclinical and clinical studies. We discuss data on the immunogenicity and efficacy of some of these vaccines.

## 1. Introduction

In recent years, viral vectors have become widely used in the development of new vaccines. This is due, first, to the high immunogenicity of these vectors, which mimic a natural infection and thus effectively stimulate two main arms of the adaptive immune response, as well as cell-mediated immunity. Another important factor is the high safety of viral vector vaccines compared to live attenuated vaccines. The most widely used viral vectors are derived from adenovirus, vaccinia virus, measles virus, herpes virus, and vesicular stomatitis virus. Among them, the vaccinia virus (VACV) is the only virus that was not originally a human pathogen. On the contrary, it became the first vaccine in human history directed against the smallpox virus. It is thanks to the massive worldwide vaccination with VACV that smallpox was eradicated. Studies of VACV led to the production of new strains, one of which, modified vaccinia virus Ankara (MVA), has been widely used as a vaccine vector since, along with the high immunogenicity typical of VACV, it had a high level of safety. The origin, properties, and application of this vector are described in this review.

## 2. History of the Origin of the Vaccinia Strain MVA and Its Properties

Until the 1960s, strains of VACV, which varied greatly in their biological properties, were used in different countries for smallpox vaccination [1]. The first strains of VACV were named after the health care institution, country, or area of origin. The most widely used strains are shown in Table 1 which was adapted from Sanchez-Sampredo et al [2]. Detailed information about the history of smallpox and the origin of VACV can be found in the study by Kaynarcalidan et al. [3].

For the preparation of the first-generation vaccine, a live virus produced on the skin of calves was used [4], so there was a high probability of microbial contamination of the preparations. In addition, such vaccines contained animal proteins, which often caused allergies in patients. Later, the cultivation of vaccine strains began to be carried out on the chorioallantoic membrane of chicken embryos or in various cell lines. This method led to the emergence of second-generation vaccines [2].

Numerous studies have shown that the use of cell cultures to grow viruses allowed for better control of vaccine production. This led to the stable production of the virus and an increase in the purity of the vaccine preparation due to the elimination of contamination by bacteria and animal proteins. However, the frequency of side effects that are characteristic of viral infections (e.g., fever, headache, malaise, and muscle aches) remained high with the second-generation vaccines. In addition, in rare cases, severe side effects such as post-vaccination encephalitis, myocarditis, and pericarditis, which required hospitalization and could lead to death, were observed [2,5,6]. Given the poor safety profile of the second-generation VACV vaccines, efforts have been made to improve their safety, leading to the third-generation vaccines [7].

Multiple passaging of the parental vaccine strain has been used to generate random mutations and deletions, which in turn have led to the attenuation of VACV and the emergence of new strains such as Lister-16m8 (LC16m8) [8], Dairen I (DIs) [9], M65 and M101 [10], NYVAC [11], ACAM3000 [4], and modified vaccinia virus Ankara (MVA) [12]. The origins of these strains and their genetic features are described in detail in the review by Kaynarcalidan et al. [3]. Of all the strains, only MVA and NYVAC are unable to replicate in human cells [13].

MVA was developed by Anton Mayr and Eberhard Munz in the 1960s as a result of sequential infection of primary chicken embryo fibroblasts (PEFs) with the chorioallantois VACV Ankara (CVA) vaccine strain, which was used for smallpox vaccination in Turkey and Germany [14]. After 516 passages, a highly attenuated laboratory virus was obtained and given the name MVA. The new virus differed from the parental CVA by the phenotype of the virus-infected chicken embryo cells. As a result of CVA infection, the fusion of squamous cells occurred, and continuous lysis was observed. In the case of MVA infection, infected cells took on a spherical shape, and the formation of individual plaques (limited areas with lysed cells) was observed. Most importantly, during the process of attenuation, MVA completely lost its ability to replicate in mammalian cells (with the exception of the Syrian hamster fibroblast cell line (BHK-21)) [2,15,16]. It is the inability to replicate in mammalian cells that distinguishes MVA from the other third-generation vaccinia strains; thus, MVA is currently considered one of the safest strains of the vaccinia virus.

**Figure 1:**
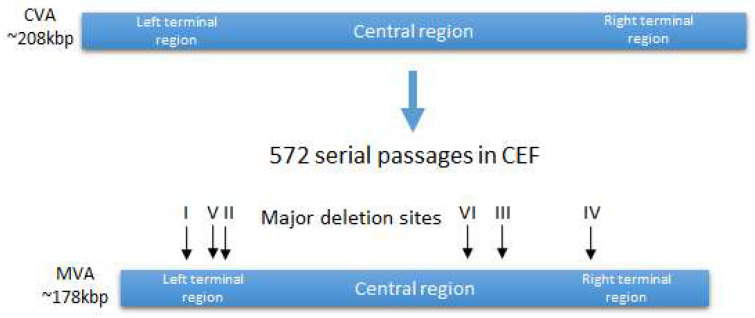
Generation of MVA strain from CVA strain. Location of the major deletion sites.

Genomic studies have shown that the parental CVA strain lost approximately 15% of its genome [12], reducing the genome length from 208 kb in CVA to 177 kb in MVA [17]. Six large genomic deletions were identified, ranging in length from 2.6 kb to 10.2 kb, as well as many shorter deletions, insertions, and point mutations, leading to fragmentation, truncation, or deletion of open reading frames (ORFs) (Figure 1) [18]. As a result of these modifications, MVA ceased to encode many virulence factors, including factors that suppress the immune response to the vaccinia virus, such as viral receptors for γ-interferon, α/β interferons, and CC chemokines [19].

In 2019 [20] and 2020 [21], restoration of the C12L and C16L/B22R genes was identified as necessary to restore the ability of MVA to productively infect mammalian cells. The functions of these genes have not yet been studied enough, but it is known that they affect the synthesis and processing of the late structural proteins of the virus. In the absence of these genes in human and other mammalian cells, the virus replicates its DNA and has undisturbed expression of early and intermediate genes, as well as of most of the late genes, but further development is blocked at the stage of virion assembly [18], which makes it impossible to form infectious progeny [22,23,24,25].

## 3. Safety of Smallpox Vaccines Based on the MVA Strain

A smallpox vaccine based on the MVA strain was first used in Germany in 1977. Due to its high safety profile, it was used for children, the elderly, and people with weakened immune systems [1,26]. In just a few years, more than 120,000 people were vaccinated.

Vaccinated patients experienced mild local reactions, such as redness at the injection site, but did not develop blisters, pustules, or ulcers, as was observed with the second-generation smallpox vaccines [27,28,29,30]. In 2.3% of cases, vaccination caused a fever, and in 4.1% of cases, it led to the development of other non-specific systemic reactions [26], but no severe side effects were detected. Since vaccination was carried out at a time when smallpox was no longer present in Germany, its effectiveness remained unexplored [31].

Another smallpox vaccine based on the MVA strain, MVA-BN (V00083008), was licensed by the European Medicines Agency in 2013 under the brand name IMVAMUNE and by the US Food and Drug Administration (FDA) in 2019 under the brand name Jynneos [32]. According to several clinical studies in immunocompromised people, including those with atopic dermatitis or HIV infection, the MVA-BN vaccine has been shown to be completely safe and highly immunogenic [33,34,35,36,37]. The vaccine was recommended in the US and Canada in 2022 to prevent monkeypox [38]. These authorizations were based on the results obtained in the study by Earl et al. [39]. This work showed that two vaccinations with MVA-BN completely protect cynomolgus monkeys from a lethal monkeypox infection.

One of the most serious complications when using the first- and second-generation smallpox vaccines was neurotoxicity. The frequency of post-vaccination encephalitis varied depending on the vaccine strain. In the past, the heavily used Lister and Dryvax^®^ vaccines averaged 2.6 and 2.9 cases of vaccinal encephalitis per million doses, respectively [40,41]. Although the incidence of encephalitis after vaccination was extremely low, a quarter of these cases ended in death, and in another quarter, irreversible neurological disorders developed [5,42,43,44]. No cases of neurotoxicity were recorded with third-generation LC16m8 or MVA vaccines [45]. Animal studies have also demonstrated that intracerebral inoculation of MVA does not lead to encephalitis, and, moreover, immunization with MVA prevents the risk of developing encephalitis after vaccination with a replication-competent vaccine [16,46,47].

The most commonly reported severe adverse events with the first- and second-generation smallpox vaccines were myocarditis and pericarditis, which occurred at a rate of 120 cases per million [48,49,50]. The MVA strain vaccine has not been shown to increase the risk of myo- or pericarditis [36,51,52].

Moreover, preliminary vaccination with MVA has been shown to be able to attenuate skin lesions caused by the first-generation Dryvax vaccine based on the replication-competent vaccinia strain NYCBOH.

It was further shown that the safety of the MVA virus depends on its homogeneity. MVA obtained by Mayr [15] has long been considered incapable of replication in mammalian cells [12,23]. However, in 2009, new data showed that at least some MVA strains deposited in different collections, such as MVA-572 (ECACC), MVA-I721 (National Collection of Cultures of Microorganisms, CNCM, Pasteur Institute Paris), MVA VR-1508 (ATCC), are heterogeneous and contain virus variants that can replicate in human cell lines and even cause lethality in immunodeficient mice [28,53,54]. At the same time, it was shown that the MVA-BN strain, obtained as a result of six rounds of purification of the MVA-584 strain by the selection of individual plaques (viral clones), was not able to replicate in any of the human cell lines considered in the study or in mice with suppressed immunity [53]. The obtained data indicate the need for a careful assessment of the homogeneity of those strains that are planned for use in clinical practice. 

Furthermore, there were concerns about the possible restoration of MVA replication as a result of its recombination with circulating orthopoxviruses in vivo, for example, when vaccinating animals. This situation was simulated in vitro by infecting cells permissive to MVA with replication-competent Norwegian vaccinia strain No-H1 simultaneously with MVA [55]. A hybrid MVA that could propagate in human cells could only be obtained by infecting the cell line with high doses of both viruses (multiplicity of infection = 5), an extremely unlikely event in vivo. It should be noted that no occurrence of a replication-competent MVA strain has been reported in any preclinical or clinical study [56].

## 4. Recombinant Vaccines Based on MVA

In addition to being used as a smallpox vaccine, the MVA virus can also serve as a vector for vaccines against other pathogens. MVA is considered a promising viral vector due to its ability to incorporate up to 25 kb of foreign DNA into its genome, to express a wide range of transgenes with correct post-translational modification, due to its high immunogenicity in vivo [57,58], and also because of its safety profile.

To date, a large number of candidate vaccines have been developed based on the MVA vector, including vaccines against HIV [59,60], tuberculosis [61], malaria [62,63,64], Ebola [65,66,67,68,69], RSV [70,71], MERS [72], CMV [73], and influenza [74,75,76], which are being studied in the late stages of clinical trials. Significantly more candidate vaccines against a variety of other human diseases appear in preclinical studies [2,18,77]. MVA is also an attractive and efficient viral vector for the development of recombinant veterinary vaccines [78,79,80,81,82,83,84,85,86,87].

## 5. Immunogenicity and Efficiency of Vaccines Based on MVA Vector

An important indicator of the effectiveness of a vaccine is its immunogenicity, i.e., the ability to induce the formation of protective antibodies and T-cell responses to the antigens of the pathogen.

The sheer volume of work related to the development of MVA-based vaccines cannot be covered in a single review, so attention will only be given to those vaccines for which clinical trial data are available or when a protective effect has been demonstrated in preclinical studies.

One infection for which attempts to develop a vaccine have been made for a long time is that caused by the human immunodeficiency virus (HIV). In particular, a vaccine called MVA-B was generated using an MVA vector, which included the monomeric envelope protein gp120 and three Gag-Pol-Nef (GPN) viral proteins of subtype B virus fused together as HIV antigens. During phase 1 of a clinical trial, participants (HIV-uninfected) received the vaccine at a dose of 10^8^ PFU three times, at intervals of 4 and 16 weeks after the first. The study of the humoral response showed that the maximum titer of antibodies was achieved 18 weeks after the first administration of the vaccine. At this time, the response was detected in 95.8% of participants. By week 48, the percentage of seropositive individuals dropped to 72.7%. At the same time point, the percentage of patients with neutralizing antibodies was 54% [85,86]. T-cell responses, as measured by ELISpot, were detected in 68% (15/22) of participants at 20 weeks post-vaccination and persisted for at least 1 year. In 13 subjects, a more detailed study of the T-cell response was performed and found that CD4+ T-cell responses mainly occurred in the Env protein, while CD8+ - T-cell responses to all vaccine antigens (Env, Gag, Pol, and Nef) were detected [88,89].

The same vaccine was administered to HIV-infected patients in another clinical trial [90]. The main goal of vaccination was to eliminate the viral reservoir by activating the T-cell response. For all patients, a significant increase in the percentage of T cells responding to the Gag antigen with the production of interferon was shown, the number of which was measured using the ELISpot assay. To study the vaccination efficiency, antiretroviral therapy was interrupted. In vaccinated patients, an increase in the time to viral rebound after antiretroviral treatment interruption was noted compared with placebo, which indicated a short-term effect of vaccination on the control of viral replication. However, vaccination failed to reduce the viral reservoir. There can be many reasons, including the high genetic variability of HIV, a problem that remains unresolved in the development of an HIV vaccine [90].

In general, both of these studies demonstrate the high immunogenicity of the vaccine. The absence of a significant effect is largely due to the pathogen itself, particularly its features, which, despite many years of efforts, do not yet allow the development of an effective vaccine. Importantly, the second study also showed the safety of using MVA in immunocompromised HIV-infected patients.

The MVA vector has also been used to create a vaccine against respiratory syncytial virus (RSV), which is dangerous for children and the elderly. Several studies have shown that vaccination with recombinant MVA carrying one or more RSV viral proteins can completely protect cotton rats susceptible to RSV infection [91,92]. In clinical trials, the MVA-BN-RSV vaccine was studied, which is a recombinant MVA that includes five antigens: surface protein F (fusion protein), two surface proteins G (glycoprotein) of different subtypes (subtype A and subtype B), and two internal proteins, N and M2.

The phase 1 clinical trial investigated two doses of the vaccine (10^7^ and 10^8^ TCID 50) given to adult participants twice, 4 weeks apart. Antibodies specific to RSV were detected in the blood serum of all participants prior to the study due to the high prevalence of the virus in the human population, and the vaccine caused a moderate increase in antibody titers to RSV of both subtypes (A and B). In the vaccine-induced T-cell response against all antigens, the percentage of activated lymphocytes increased 1.8–3.8 times compared with the values obtained before vaccination [93]. Cellular response to three or more antigens was observed more often in participants who received the highest dose (83% compared to 63% in the case of a low dose).

Additionally, the safety and immunogenicity of the vaccine were studied in a group of people over 55 years of age. Two doses of 1 × 10^8^ and 5 × 10^8^ TCID50 were studied. Participants were divided into four groups, which were administered one or the other dose once or twice with an interval of 4 weeks [71]. The incidence of side effects increased with increasing dose, but no serious side effects were found in any group. The dose of 5 × 10^8^ TCID50 caused a stronger humoral and T-cell response; no significant differences were found between single or double administration. After the first high dose immunization (5 × 10^8^ TCID50), the level of neutralizing antibodies and the levels of IgG and IgA RSV-binding antibodies increased 1.6–3.4 times compared to baseline. T-cell response to all five antigens that are part of the MVA-BN-RSV was detected. An increase in the number of cells responding to antigens by 5.4–9.7 times was noted. Antibodies remained above baseline for 6 months, and T-cell response remained above baseline for 1 year. A booster dose of the vaccine given 12 months later re-elevated antibody levels and specific T-cell levels. In April 2022, the phase three clinical trial was initiated, which aims to enroll 20,000 people over 60 years of age.

The MVA vector has been used in the development of several so-called universal influenza vaccines, aiming to protect against a wide range of viral strains. One such vaccine was designed to protect against avian influenza viruses and contained hemagglutinins from three different strains of H5N1 avian influenza virus: Vietnam/1203/04, A/Indonesia/CDC669/06, and A/Anhui/01/05 [94]. In balb/c mice, it was shown that the vaccine induced the production of a high titer of antibodies that inhibited hemagglutination at dilutions of 1:480, 1:510, and 1:350 against the viruses included in the vaccine, as well as at dilutions of 1:290, 1:340, and 1:270 against heterologous viruses from clades four, seven, and nine. The titers of protective antibodies determined by microneutralization correlated with data on hemagglutination inhibition. The vaccine provided 100% protection to mice against lethal challenges with homologous clade 1 H5N1 virus (RG-A/Vietnam/1203/04) or heterologous clade seven virus (RG-A/chicken/Shanxi/2/06).

Another influenza vaccine, MVA-NP + M1 (VTP-100, from Vaccitech), has been designed to generate a T-cell response against highly conserved internal antigens in order to provide protection against a wide variety of influenza viruses. The vaccine is a recombinant MVA containing the NP nucleoprotein and the M1 matrix protein fused into a single polypeptide (NP + M1). This vaccine has been studied in a large number of clinical trials.

In a phase one clinical trial, it was shown that a single intramuscular dose of 2.5 × 10^8^ PFU caused an increase in T-cell response, measured using the ELISpot assay. After vaccination, the number of cells secreting IFN-γ in response to antigen stimulation increased more than 10-fold [95]. Another phase two clinical trial investigated the efficacy and safety of the same vaccine at a dose of 1.5 × 10^8^ PFU. At 21 days after a single injection, the vaccinated showed an increase in the T-cell response by more than three times. The uniqueness of this study is that participants were artificially infected with the influenza virus on day 22 after vaccination to assess the protective effect of the vaccine. The study involved 22 volunteers, of whom 11 were vaccinated with MVA-NP + M1, and the other 11 received a placebo. Influenza virus A/Wisconsin/67/2005 was used for infection. As a result, two participants in the vaccinated group and five in the control group developed laboratory-confirmed influenza. Flu symptoms in participants who received the vaccine were less pronounced. There was also a reduction in the duration of virus shedding from the nasopharynx in patients with influenza in the vaccinated group compared with the control group [76]. The vaccine has been studied in several other clinical trials for different age groups, both as a single vaccination and in combination with a seasonal vaccine, and even for heterologous vaccination with AdV [96]. In all studies, the introduction of MVA-NP + M1 was accompanied by a significant increase in the number of T cells specific for influenza virus antigens. However, despite a significant increase in the T-cell response, two phase 2 studies aimed at studying the effectiveness of the vaccine showed no protection against infection (NCT03880474, NCT03883113). These data indicate the need for further research on the role of the T-cell response in protection against the influenza virus, including the study of the activation of different T-cell populations during infection and vaccination. It is also possible that in the case of influenza, the formation of only a T-cell response is not enough, and effective protection is possible only in the case of interaction between the humoral and T-cell response.

Our research group is also developing a universal influenza vaccine using an MVA vector. This vaccine contains epitopes from conserved regions of the NP, M1, and HA proteins of influenza A and B, which were selected as antigens using our own algorithms. Double immunization with the vaccine protected mice with a 67% or greater efficiency against lethal challenges with strains of the H1N1, H2N2, H3N2, and H5N1 subtypes of influenza A [97].

Of great interest is the development of vaccines against coronaviruses. The first attempts to use MVA as a viral vector were made to create a vaccine against the Middle East syndrome virus MERS-CoV, which causes severe lung disease with a high mortality rate in humans. To create a vaccine against this virus, the full-length envelope protein S was used as an antigen. The immunogenicity and protective efficacy of the vaccine were studied in mice that were intranasally transduced with an adenoviral vector encoding the human dipeptidyl peptidase 4 receptor, which is a cellular receptor for the MERS-CoV virus. The MVA-MERS-S vaccine caused the formation of virus-neutralizing antibodies and virus-specific CD8+ cells. The vaccine effectively protected animals from MERS-CoV infection: on the fourth day after infection, the virus was not detected in the lungs, and histopathological analysis showed no damage to the lungs [98]. Phase one clinical trials were conducted and showed that after immunizations with two doses of 10^7^ or 10^8^ PFU, neutralizing antibodies were generated in 75% and 82% of participants, respectively [69]. Furthermore, vaccination led to the development of a T-cell response.

Another example of an MVA-based vaccine under development is the vaccine against the SARS-CoV-2 pandemic virus. To create it, two full-length viral proteins were chosen as antigens: the envelope protein S and the internal nucleocapsid protein N [99]. In preclinical studies in mice, the vaccine was administered twice, 21 days apart. The vaccine stimulated a good humoral response with a high titer of neutralizing antibodies. A T-cell CD8+ response was also detected: the percentage of cells producing IFN-γ and TNFα in response to antigen stimulation was 3% and 7%, respectively.

The effectiveness of developing a humoral response, including neutralizing antibodies, was also demonstrated in Syrian hamsters, with a double injection of the same vaccine at a dose of 10^8^ PFU. Vaccination protected hamsters from developing infection and prevented virus replication in the lungs and weight loss in animals after SARS-CoV-2 infection [100].

The vaccine candidate was also studied in non-human apes. African green monkeys were vaccinated either twice with a dose of 2.5 × 10^8^ PFU 4 weeks apart or once with a dose of 5 × 10^8^ PFU. Three weeks after the last vaccination, high titers of antibodies to S and N proteins were detected in all vaccinated animals. Determination of neutralizing antibodies showed that two vaccinations provided a higher titer. Analysis of the T-cell response using the ELISpot assay showed the presence of S- and N-specific T-cells that secreted interferon and IL-2 in response to antigen stimulation. With two vaccinations, a stronger T-cell response to the S-protein was observed. Cellular response to N-protein was weaker and did not differ between groups. After 6 weeks, the animals were infected with the virus using the combined intranasal/intratracheal route of infection. On the second day after infection, the viral load and infectious titer in both nasal swabs and bronchoalveolar lavage were two to three orders of magnitude lower in vaccinated monkeys than in control animals. Viral load and titer remained lower in vaccinated animals at all analyzed time points after infection. Histopathological analysis showed no foci of inflammation in the lungs and bronchi in these monkeys.

Interesting data have been obtained in preclinical animal studies of two MVA-based vaccines: against yellow fever and the Chikungunya virus [101,102]. The presence of a lethal mouse model in both cases made it possible to demonstrate the high protective effect of these vaccines. At the same time, passive transfer of serum from vaccinated to unvaccinated mice did not protect the animals, indicating that the humoral response did not play a decisive role. It was concluded that the T-cell response induced by vaccination was the crucial factor, and an increase in the proportion of T-cells recognizing viral antigens was shown.

In the studies of MVA-based vaccines described above, vaccination was carried out using the most common route of administration—intramuscular. This route of immunization leads to the induction of systemic immunity. However, local administration of vaccines in the respiratory tract may be promising, as it can contribute to the formation of local mucosal immunity, which is an important first-line defense against airborne viruses. In addition, immunization targeting local mucosal surfaces is less invasive and, therefore, may be more widely used.

The mucosal route of administration of vaccines against SARS-CoV-2 and influenza viruses was studied by several research groups [79,103,104,105,106,107,108,109]. It was demonstrated that, in contrast to intramuscular administration, mucosal administration of MVA-based vaccines to animals caused effective production of IgA antibodies and the formation of antigen-specific T-cells locally in the studied organs. At the same time, the data on the formation of a systemic humoral response (the formation of IgG in the blood serum) were controversial. There is data showing that both intranasal and IM administration efficiently stimulate the production of IgG [103,104]. Another study [105] has demonstrated that the IgG titer was an order of magnitude lower after mucosal immunization than after intramuscular immunization. Finally, in the work of Zhong et al. [106], antibodies in serum were not detected at all after mucosal immunization. Additionally, in a clinical study of candidate TB vaccine MVA85A, no IgG antibodies to Ag85A TB antigen were observed when using aerosol immunization [107,108].

The mucosal immunization with MVA-based vaccines was shown to protect animals from infection [103,104,106]. Thus, Jeffrey et al. [104] studied a candidate vaccine against SARS-CoV-2, which is a recombinant MVA containing a modified S protein. Transgenic K18-hACE2 mice susceptible to SARS-CoV-2 were vaccinated twice with a dose of 2 × 10^7^ PFU intramuscularly or intranasally and challenged with SARS-Cov-2 virus 2 weeks later. Both intranasal (IN) and intramuscular (IM) administration of the vaccine completely prevented morbidity. However, the authors believe that IN vaccination was more effective since it not only provided rapid elimination of the virus from the upper respiratory tract, as in the case of IM vaccination but also completely prevented virus replication in studied tissues. In the study by Moss et al. [109], MVA expressing influenza HA and NP proteins (MVA-HA-NP) was used for a single-dose vaccination of mice via either the IM or IN route. IM administration of a dose of 1 × 10^5^ infectious units protected all mice against a challenge with the lethal dose of influenza virus, while administration of 1 × 10^6^ intranasally prevented the death of only 75% of mice, indicating that the dose for mucosal vaccination should be higher. In another study of the same MVA-HA-NP construct [105], double intragastric immunization of animals with a dose of 1 × 10^8^ PFU completely protected mice against homologous influenza virus challenge. Alternation of vaccine administration routes when using the prime-boost vaccination was also studied. Prime intramuscular and boost intranasal administration was shown to be effective. This scheme led to the formation of both local and systemic immunity, as well as effective protection against infection [79,103,104]. Such heterologous administration may be preferable because it can provide systemic immunity more effectively. It is necessary, for example, in the case of SARS-CoV-2 infection, which is not restricted to the respiratory system but can affect other organs. At the same time, the safety of this approach should be evaluated. Thus, despite the proven safety profile of MVA when administered intramuscularly, transient moderate/severe respiratory and systemic adverse effects were observed in volunteers who received aerosol tuberculosis MVA85A vaccine as a booster [107]. Due to the safety profile, the second dose was canceled for the last three participants.

The above examples suggest that local administration of MVA-based vaccines can be very promising. However, further comprehensive studies are needed to elucidate the duration of emerging immunity, the safety and efficacy of this route in the clinic, and the effective human dose. 

## 6. Preexisting Vector Immunity

The presence of pre-existing immunity to the vaccinia vector can adversely affect the efficacy of MVA-based vaccines. Such immunity can lead, on the one hand, to accelerated elimination of the vector from the body, which will interfere with the formation of a response against the delivered target antigen, and, on the other hand, to increased side effects associated with a violent reaction of the immune system to the vector.

This problem is relevant for older people who have immunity to vaccinia due to their participation in vaccination against smallpox. However, several clinical trials of recombinant MVA-based vaccines have shown that previous vaccination against smallpox had no effect on the development of an immune response to an MVA-administered antigen [110,111].

Furthermore, the problem of reducing the effectiveness of immunization associated with the formation of an immune response to the vector can potentially arise when a vaccine based on the same vector is repeatedly administered to people, even if they have not been vaccinated against smallpox. Several clinical studies have shown that repeated injections of recombinant MVA vaccine do not prevent the activation of the humoral response and allow an increase in the antibody titer to the target antigen [74,112,113]. At the same time, data on the effect of repeated vaccination using the MVA vector on the effectiveness of the formation of T-cell immunity were contradictory. Along with the studies showing that the first vaccination does not affect the re-induction of a T-cell response when using the same vector [113], there are published studies reporting that the cellular response after re-vaccination was decreased [114].

In addition to the impact of re-vaccination on efficacy, there were concerns about the potential for increased side effects after the re-introduction of the vector vaccine. However, clinical studies have shown that, on the contrary, side effects after the second immunization were less pronounced than after the first dose [72]. 

To avoid the re-introduction of the vector, different vectors carrying the same genes can be used. Such a vaccination is called heterologous vaccination. Several studies in animal models compared the efficacy of homologous vaccination using the MVA vector alone with heterologous vaccination where the same antigen was delivered 4 weeks apart using a DNA vector or adenovirus vector (AdV) and also as a protein. It has been demonstrated that administration of rMVA-S encoding the S antigen of SARS-CoV followed by AdV-S vector with the same antigen leads to the production of higher titers of neutralizing antibodies and increases the time of their persistence compared with two vaccinations with the rMVA-S in mice and rabbits [115]. In another study, titers of neutralizing antibodies against cytomegalovirus antigen were higher with sequential vaccination with protein antigens and a recombinant MVA-PC vector than with homologous vaccination with MVA-PC alone [116]. In addition, in the study of the malaria vaccine, it was shown that the T-cell response after heterologous vaccination using a DNA vector or an AdV followed by the introduction of the MVA vector was two or more times higher compared with homologous vaccination using only the MVA vector for antigen delivery. An increase in the T-cell response led to increased protection against malaria [117,118]. Thus, under certain conditions (correct selection of vectors, doses, antigens, routes of administration, etc.), heterologous vaccination can be more effective than homologous.

Heterologous vaccination using MVA as one of the vectors has also been investigated in clinical trials, although comparisons with homologous vaccination have not been made. Among the works, we can mention the study of heterologous vaccines against influenza virus [119], HIV [120], Ebola virus [121,122,123], and malaria [124], for which a good humoral and/or T-cell response was shown. A detailed discussion of such studies is beyond the scope of this review.

## 7. Conclusions

The data obtained in preclinical studies and clinical trials indicate the high immunogenicity of MVA-based vaccines. The most commonly used vaccine dose of 10^8^ PFU allows the induction of both humoral and T-cell responses. It should be noted that in order to produce neutralizing antibodies, which are the most effective for protection and can provide sterile immunity, it is necessary to use the surface proteins of pathogens as an antigen. However, this strategy does not work well for highly variable viruses, such as the HIV or influenza viruses, because their surface proteins differ greatly between strains. In this case, T-cell immunity comes to the fore. The T-cell response is formed as a response to both external and internal proteins of the virus, which are usually more conserved, and thus are able to provide some protection (but usually not sterile immunity) against a wide range of pathogen strains. Therefore, one of the important advantages of MVA-based vaccines is the ability to activate both arms of immunity, which makes this platform universal and allows it to achieve more effective protection. The high capacity of the MVA vector allows the insertion of more than one foreign antigen, so both surface and internal antigens can be included.

Additionally, the potential of the MVA-based vaccine vector can be increased by modifying its genome. Targeted genetic manipulations within the VACV genome aimed at improving its immunogenicity and vaccine performance have been reviewed elsewhere [3]. 

Separately, there is the issue of the effectiveness of MVA-based vaccines. The results obtained in clinical studies do not always demonstrate their effectiveness. It should be emphasized that the effectiveness of vector vaccines depends on many factors, among which the most important and determining factor is the competent choice of antigens, so in some cases, failure may be due to the non-optimal antigen selection. It is also important to note that the vast majority of MVA-based vaccines are developed to fight the most “difficult” pathogens, against which a vaccine cannot be obtained using traditional approaches. Often, such pathogens are highly variable viruses. Approaches to address the problem of high variability are still being developed, and the creation of an effective vaccine, in this case, is not an easy task.

To date, MVA has proven itself as a vector for the development of vaccines against various infections. Attractive properties of MVA are a high safety profile, the ability to induce humoral and T-cell responses, low production costs, and the absence of special requirements for storage and transportation. This vector is of particular interest for developing vaccines for people with weakened immune systems, the elderly, or HIV-infected people, for whom the use of traditional vaccines may be associated with certain risks. Several vaccines are currently undergoing clinical trials that will provide a better understanding of prospects for their further application.

## Figures and Tables

**Table 1:** List of the first VACV strains used for vaccination [2].

VACV Strain	Country or Region of Application
New York City Board of Health (NYCBOH), Dryvax	USA
Lister	UK, Europe, Asia, Africa, USA
Temple of Heaven (Tian Tan)	China
Tashkent, Gam, MRIVP, Per, B-51	USSR
Lister/L-IVP	USSR/Russian Federation
Bern	Germany, Austria
Paris	France, Syria, Turkey
Copenhagen	Denmark
Dairen, Ikeda	Japan
Sweden	Sweden
Ankara	Turkey
Chambon	France and Africa

## Data Availability

Data are available from the authors upon request (O.V.O).
